# Innate/Inflammatory Bioregulation of Surfactant Protein D Alleviates Rat Osteoarthritis by Inhibiting Toll-Like Receptor 4 Signaling

**DOI:** 10.3389/fimmu.2022.913901

**Published:** 2022-07-05

**Authors:** Huanyu Jiang, Yubiao Zhang, Geliang Hu, Xiaobin Shang, Jianghua Ming, Ming Deng, Yaming Li, Yonggang Ma, Shiqing Liu, Yan Zhou

**Affiliations:** ^1^Department of Orthopedics, Renmin Hospital of Wuhan University, Wuhan, China; ^2^Central Laboratory, Renmin Hospital of Wuhan University, Wuhan, China

**Keywords:** surfactant protein D, osteoarthritis, inflammation, innate immunity, chondrocyte, toll-like receptor 4

## Abstract

Osteoarthritis (OA) is a deteriorating disease of cartilage tissues mainly characterized as low-grade inflammation of the joint. Innate immune molecule surfactant protein D (SP-D) is a member of collectin family of collagenous Ca^2+^-dependent defense lectins and plays a vital role in the inflammatory and innate immune responses. The present study investigated the SP-D-mediated innate/inflammatory bioregulation in OA and explored the underlying molecular mechanism. Transcriptome analysis revealed that SP-D regulated genes were strongly enriched in the inflammatory response, immune response, cellular response to lipopolysaccharide (LPS), PI3K-Akt signaling, Toll-like receptor (TLR) signaling, and extracellular matrix (ECM)-receptor interaction pathways. Knockdown of the SP-D gene by the recombinant adeno-associated virus promoted the macrophage specific markers of CD68, F4/80 and TLR4 in the articular cartilage *in vivo*. SP-D alleviated the infiltration of synovial macrophages and neutrophils, and inhibited TLR4, TNF-α and the phosphorylation of PI3K, Akt and NF-κB p65 in cartilage. SP-D suppressed cartilage degeneration, inflammatory and immune responses in the rat OA model, whilst TAK-242 strengthened this improvement. In *in vitro* conditions, SP-D pre-treatment inhibited LPS-induced overproduction of inflammation-correlated cytokines such as IL-1β and TNF-α, and suppressed the overexpression of TLR4, MD-2 and NLRP3. SP-D prevented the LPS-induced degradation of ECM by down-regulating MMP-13 and up-regulating collagen II. Blocking of TLR4 by TAK-242 further enhanced these manifestations. We also demonstrated that SP-D binds to the TLR4/MD-2 complex to suppress TLR4-mediated PI3K/Akt and NF-κB signaling activation in chondrocytes. Taken together, these findings indicate that SP-D has chondroprotective properties dependent on TLR4-mediated PI3K/Akt and NF-κB signaling and that SP-D has an optimal bioregulatory effect on the inflammatory and innate responses in OA.

## Introduction

Osteoarthritis (OA) is a deteriorating disease of cartilage tissues, and inflammation plays a leading role in its pathogenesis (1). The pathogenesis of OA involves not only the destruction of cartilage tissues but also the remodeling of subchondral bones, formation of ectopic bones, articular cartilage hypertrophy, and inflammation of the synovial lining (2). OA affects the whole joint and causes inflammation and other clinical manifestations. The inflammation in OA is chronic, relatively mild, and mediated primarily by the innate immune system (3).

Surfactant protein D (SP-D) is a member of the soluble C-type lectin family called collectins. This protein acts as a link between innate immunity and adaptive immunity and prevents infection, allergy, and inflammation (4, 5). The lung stabilizing effect of SP-D is widely known. Still, its stabilizing roles in extra-pulmonary tissues such as articular cartilage, brain, testes, heart, kidneys, and pancreas are poorly understood. In addition, SP-D is expressed at different levels in the synovial fluid of rheumatoid arthritis patients and is involved in the pathogenesis of rheumatoid arthritis (6–8). Our previous studies have shown that SP-D is highly expressed in the cartilage and regulates chondrocyte apoptosis (9, 10).

SP-D modulates the immune function by interacting with Toll-like receptors (TLRs), which are a type of pattern recognition receptors (PRRs) (11, 12). TLR4 activates the innate immune response by recognizing danger-associated molecular patterns (DAMPs) that are mainly endogenous signals for cell death and tissue damage. Lipopolysaccharide (LPS), an outer surface component of Gram-negative bacteria, is an exogenous TLR4 agonist, while high mobility group box 1 and heat shock proteins are endogenous TLR4 agonists (13). Opioid-induced non-stereoselective activation of TLR4 and increased activation of TLR4 signaling by the release of DAMPs, may act as a critical trigger for continuous NOD-like receptor protein 3 (NLRP3) inflammasome activation (14). Studies have shown that SP-D can inhibit the activation of alveolar macrophages and dendritic cells *via* mite allergen-induced TLR4 signaling. The inhibition by SP-D depends on the binding of carbohydrate recognition domain (CRD) to the extracellular domain of TLR4 (15). OA-associated inflammation has been linked to the innate immune response *via* different mechanisms including the activation of TLRs (16). TLRs are members of a highly conserved family of receptors that recognize either pathogen or DAMPs, which are host-derived molecules released as a response to tissue stress and injury (17). TLR4 is involved in the production of innate immune factors that increase synovitis, cartilage degradation and osteoarthritis (18).

In this study, we explored the functional relevance of SP-D to better understand its role as a suppressor of OA-associated immune responses and inflammation in chondrocytes. We further analyzed the impact of SP-D on the signal transmission potential of the TLR4-mediated signaling in osteoarthritic chondrocytes. All these findings suggest that SP-D may be a potential target for OA treatment.

## Materials and Methods

### Reagents and Antibodies

F4/80 (#SAB5500103) was obtained from Sigma-Aldrich (St. Louis, MO, USA). The TLR4 inhibitor TAK-242 (#614316) was obtained from Calbiochem (San Diego, CA, USA). The PI3K inhibitor wortmannin (#HY-10197) was obtained from MedChemExpress (New Jersey, USA). Collagen II (#28459-1-AP) was obtained from Proteintech Group (Wuhan, China). TLR4 (#AF7017), CD68 (#DF7518), PI3K (#DF6069) antibodies were obtained from Affinity Biosciences (Cincinnati, OH, USA). SP-D (#ab220422), MD-2 (#ab24182), TNF-α (#ab66579), glyceraldehyde-3-phosphate dehydrogenase (GAPDH) (#ab181602), MMP-13 (#ab84594), and NLRP3 (ab263899) antibodies were obtained from Abcam (Cambridge, UK). Antibodies for NF-κB p65 (#8242), phospho-p65 (p-p65) (#3033), p-Akt (#4060) and Akt (#9272) were procured from Cell Signaling Technology (Boston, MA, USA). Recombinant human SP-D (rhSP-D) (#CSB-YP021175HU) was purchased from Huamei Biotech Co., Ltd (Wuhan, China). ELISA Kits of rat IL-1β (#ELK1272) and TNF-α (#ELK1396) were purchased from ELK Biotechnology (Wuhan, China). SNP (#1008) was purchased from Youcare Pharmaceutical Group Co., Ltd (Beijing, China). All of the other chemicals and reagents were of analytical grade.

### Cell Culture, RNA Isolation and Sample Preparation

Primary chondrocytes were isolated from the knee joint of Sprague-Dawley (SD) newborn rats. The 0.5-1 mm^3^ pieces of cartilage were digested with 0.25% trypsin and 0.02% EDTA for 1 h. The samples were transferred to a dish containing 0.2% type II collagenase and incubated for 4 - 5 h at 37°C. The suspended cells were carefully collected by pipette aspiration. Cells were washed and resuspended in complete Dulbecco’s modified Eagle’s medium/F12 (Hyclone, USA), supplemented with 10% fetal bovine serum and penicillin/streptomycin. The primary cells were frozen and stored for subsequent cell experiments.

Third generation of rat chondrocytes were seeded in 6-well culture plates for 12 h. The complete cDNA length of the SP-D gene was cloned into the pcDNA3.1 vector (Youbio Biotech Changsha, PRC) using the hot fusion method designed with CE Design V1.04 (Vazyme Biotech Co., Ltd). Each primer comprises of a fragment of gene specific sequence and a 17-30 bp sequence of the pcDNA3.1 vector. This vector also harbored a FLAG tag (Sigma), which was fused to the 3’ end of SP-D and used as a labeled protein. After the cells grew to about 80% confluence, the medium was removed, and the cells were co-treated by lipofectamine transfected pcDNA3.1 empty plasmid and pcDNA3.1-SP-D plasmid (1 μg/μL). The media was changed after 6 h, and the samples were collected at 48 h. The culture medium was removed at the specified time point, and the total RNA of cartilage tissue cells was isolated using the GenElute™ Mammalian Total RNA Miniprep kit (Sigma). The total RNA was resolved with DNAse I (Qiagen, Hilden, Germany). The concentration and consistency of RNA were determined with a 2100 Microbial Detector (Agilent Technologies). TaqMan reverse transcription reagents and a hexamer (Applied Biosystems, Foster City, CA, USA) were used to reverse transcribe the RNA (150 ng/test sample) into cDNA for RT-PCR.

### RNA-Sequencing and Bioinformatics Analysis

RNA-Sequencing was carried out using RNA samples to ensure sufficient RNA quantity and less variation. The process involved total RNA isolation, cDNA library preparation, and RNA transcriptome sequencing (Illumina HiSeq 4000). These procedures were carried out by Shenzhen BGI Tech Co., Ltd. Both pcDNA3.1-SP-D and control chondrocytes had three biological replicates. There were six RNA-seq samples (SP-D_1st, SP-D_2nd, SP-D_3rd, Ctrl_1st, Ctrl_2nd, and Ctrl_3rd). The fragments per kilobase of transcript per million fragments mapped (FPKM) was used to present the level of gene expression. The features of RNA were verified by Agilent 2100 Microbial Detector (Labx, Midland, Canada). During RNA-sequencing, a template with RNA Concordance Number > 6.5 was used. After sequencing the transcriptome, low-quality, environmental pollutants from the power adapter and high-component unidentified base (N) noise readings were filtered out. Bowtie 2 (18) (http://bowtie-bio.sourceforge.net/Bowtie2/index.html) was then used to align the reads to the reference gene (NCBI Rnor 6.0). Subsequently, DEseq2 was used to test the differential expression genes (DEGs). The fold change was > 2, and the adjusted P value was < 0.05. A heat map was created using MeV (https://sourceforge.net/projects/mev-tm4/) to assess the performance levels of DEGs. To determine the gene functions, DEGs were used as inputs to the Gene Ontology (GO) and Kyoto Encyclopedia of Genes and Genomes (KEGG) databases.

### Recombinant Adeno-Associated Virus (rAAV) Construction and Animal Studies

ShRNAs specific for SP-D/scrambled controls were cloned into the GV478 AAV vector (Shanghai Genechem Co., Ltd), and co-transfected into AAV-293 cells with pAAV-RC and pHelper vectors. rAAV particles were isolated from cell supernatants, concentrated and purified for *in vivo* studies.

Adult male Sprague-Dawley (SD) rats were procured from Wuhan University Clinical Experiment Management Center/ABSL-III laboratory. The animal studies were approved by the Federation of Small Animal Care and Application of Wuhan University Medical College. In the rAAV serotype study of small animals with normal bones and joints, rats were injected with rAAV encoded SP-D-specific shRNA intra-articularly [1×10^10^ deoxyribonuclease resistant particle (drp)/25 μl/knee joint] for 10 consecutive days (**Table 1**). The rats were sacrificed at the fourth week of the first injection, and no other intervention was carried out during this time.

**Table 1:** The treatments in each group.

Group	PBS	rAAV-GFP	rAAV-SP-D shRNA	TAK-242	rhSP-D
Control group	+	−	−	−	−
rAAV-GFP + Control group	−	+	−	−	−
rAAV-SP-D + Control group	−	−	+	−	−
Sham operation group	+	−	−	−	−
OA-induction group	+	−	−	−	−
OA + TAK-242 group	−	−	−	+	−
OA + rhSP-D group	−	−	−	−	+
OA + rhSP-D + TAK-242 group	−	−	−	+	+

All the rats were fed under the standard conditions for one week to acclimatize them to the laboratory conditions. The rats were randomly divided into five groups (n = 5): sham operation group, OA-induction group, OA + TAK-242 group, OA + rhSP-D group, and OA + rhSP-D + TAK-242 group. OA was induced through transection of the anterior cruciate ligament of the knee joint and resection of the medial meniscus (ACLT + MMx), followed by active movement of the rats in the electronic rotating cage (19, 20). The joint cavity injection dose was kept as 40 μl TAK-242 (4 μM) and 40 μg/mL rhSP-D once per week, and the administration of the dose started fourth-week post-surgery. Phosphate buffered saline (PBS) was injected in the sham operation group rats and OA induction group rats (**Table 1**). At 10 weeks post-operation, the animals were euthanized by cardiac exsanguination.

### Histological Analysis

The knee joints of the rats were separated, fixed with 4% paraformaldehyde for 24 h, decalcified for 6 weeks, and embedded with paraffin wax. 5 µm sagittal sections were prepared and stained with toluidine blue-O and hematoxylin-eosin (H&E). The pathophysiology was analyzed by two blind observers according to the modified Mankin scoring system (21). In addition, the levels of proteoglycan in the cartilage tissues were determined by Safranin-O-Fast Green staining.

### Examination of Immune Cell Infiltration in Synovial Tissues

The synovial tissue samples were collected from the side of the iliac tendon and fixed in 2.5% glutaraldehyde in 0.1 M PBS (pH 7.3). The test samples were washed with PBS, treated with 1% osmium oxide at 4°C, and immobilized for 3 h. The samples were rinsed in dH_2_O, dried with alcohol and toluene concentration gradient, and embedded in epoxy resin. The sliced sections were two-way colored and imaged with a transmission electron microscope (TEM) (HITACHI, H-600IV, Japan).

### Immunohistochemistry and Immunofluorescence Analysis

The TLR4, F4/80, TNF-α, SP-D, and CD68 expressions in articular cartilage were evaluated by immunohistochemistry, and SP reaction was carried out according to the manufacturer’s protocol. Briefly, cartilage sections were treated with a moderate primary antibody and visualized using an optical microscope. The percentage of positively immunostained cells was measured. The immunofluorescent mean densities of p-PI3K, p-Akt and p-p65 in cartilage samples were assessed by Image-Pro Plus 6.0 image analysis software (Media Cybernetics Co., USA). The tissue sections were incubated with a fluorescent-conjugated secondary antibody (Boster Biological Engineering, Wuhan, China) under dark conditions for 1 h and visualized using fluorescence microscopy (AX10, Carl Zeiss).

### Cell Stimulation

Primary chondrocytes from SD newborn rats were resuscitated and cultured. The cells were grown inside a humidified 5% CO2 incubator at 37°C and passed down until 80% converged. After reaching the third generation, cells were seeded in 6-well culture plates for 12 h, the medium was removed and pre-treated with a series of rhSP-D concentrations, pcDNA3.1-SP-D (1 μg/μL), TLR4 inhibitor TAK-242 (1 μM), and wortmannin (3 nM) for 2 h before LPS (1 μg/mL) co-treatment for 24 h.

### Western Blotting

The chondrocyte proteins were isolated using the total protein extraction kit according to the manufacturer’s instructions. The protein samples were separated by SDS-PAGE, transferred to PVDF membrane, and blocked with 5% (w/v) skimmed milk powder diluted with TBST for 1 h. The primary antibodies against collagen II, MMP-13, NLRP3, TLR4, MD-2, p65, p-p65, p-PI3K, PI3K, p-Akt, Akt, and GAPDH were then used to probe the blots at 4°C overnight. The blots were washed in TBST [50 ml TrisHCL (1 M, pH7.5), 8g Nacl, 0.2 g Kcl, 0.5 ml Tween 20, in 1L distilled water], incubated with HRP-conjugated secondary antibodies for 1 h, treated with enhanced electrochemiluminescence detection reagent (Amersham Biosciences, USA), and visualized using Odyssey infrared imager protein detection system software (LI-COR Bioscience, NE, UK). The relative expression level of the target protein was normalized to the band intensity of GAPDH.

### ELISA Analysis

The levels of IL-1β and TNF-α were determined in the culture supernatant, collected at the experimental endpoint. IL-1β and TNF-α levels were measured using rat ELISA kits according to the manufacturers’ protocol (technical duplicates were measured and mean values were used). Absorbance values were determined at 450 nm using a microplate reader and standard curves relating concentration to absorbance values were plotted.

### Immunofluorescence

The chondrocytes were allowed to grow and develop to 70% confluence on the 6-well plate and subjected to a serum protein starvation period before the test. The cells were immobilized in 4% paraformaldehyde for 20 minutes, infiltrated in 0.5% Triton X-100 for 5 minutes, and blocked in 1% BSA for 10 minutes. The cells were then incubated for 2 h with rabbit antibodies against PI3K and NF-κB p65. The cells were washed again and incubated with Cy3 coupling reaction secondary antibody (Bost Bioengineering, Wuhan, China) for 1 h. DAPI was then used for nuclear staining, and the cells were imaged using a fluorescence microscope (AX10, Carl Zeiss).

### Molecular Docking

The X-ray crystal structures of TLR4/MD-2 homodimer complex (PDB code: 3VQ2, screen resolution: 2.48 Å) were downloaded from the RCSB protein data bank (http://www.rcsb.org/). Based on the Tripos force field and G-asteiger-Huckel charge in the Sybyl package, the chemical structure of SP-D was constructed. The Surflex-Dock program flow was used to simulate the molecular interactions between SP-D and TLR4/MD-2. Finally, UCSF PyMoL was used to transform the output into a 3D image for visualization.

### Statistical Analysis

Data are presented as mean ± standard error of the mean (SEM). Student’s t-test and one-way analysis of variance (ANOVA) with Spearman rank correlation test were used for two-group and multi-group comparisons, respectively. All statistical analyses were performed by SPSS version 15.0 and GraphPad Prism 5 software (San Diego, Florida, UK). A P value of < 0.05 was considered statistically significant.

## Results

### SP-D Overexpression Broadly Affected the Gene Expression Profile of Chondrocytes

**Figure 1:**
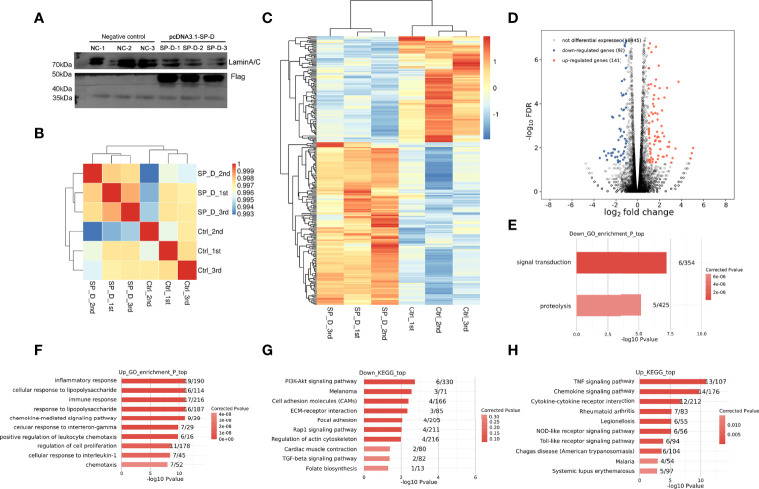
Effects of SP-D overexpression on the gene expression profile of chondrocytes. **(A)** Chondrocytes were co-treated by lipofectamine transfected pcDNA3.1 empty plasmid and pcDNA3.1-SP-D plasmid (1 μg/μL). The protein expression of SP-D was assessed *via* western blotting. **(B)** The clustering analysis of samples showed high similarity, which confirmed the correctness of the experimental design and sample sampling. **(C)** The heat map showed that the gene expression patterns and clustering relationships of the samples were similar. **(D)** Comparisons between samples showed the number of differentially expressed genes. **(E, F)** GO analysis showed that the biological processes such as regulation of ‘inflammatory responses’, ‘immune response’ and ‘response to LPS’ were associated with SP-D in chondrocytes. **(G, H)** KEGG pathway analyses revealed these genes to be significantly linked to pathways including the ‘PI3K-Akt signaling pathway’, ‘ECM-receptor interaction’, ‘TLR signaling pathway’, and ‘TNF signaling pathway’.

To explore the targets regulated by SP-D in OA, the expression of SP-D was overexpressed in chondrocytes by transfection of pcDNA3.1-SP-D plasmid. Compared with control plasmid, pcDNA3.1-SP-D effectively promoted protein expression of SP-D (**Figure 1A**). Then, RNA-Sequencing was used to detect the gene expression profiles of SP-D overexpression and control chondrocytes. The amount of the cleaned reads that were mapped to the human genome averaged 73 million per sample and the amount of uniquely mapped reads averaged 62 million (**Supplementary Table S1**).

Based on the FPKM values of each expressed gene, a correlation matrix was constructed for the six samples that were used for unsupervised hierarchical clustering. As shown, there was a clear separation of the pcDNA3.1-SP-D and control samples, with the three biological replicates clustered together (**Figure 1B**). This result demonstrated that SP-D overexpression obviously changed the gene expression profile of chondrocytes.

The differential gene cluster map showed a close relationship between the gene expression pattern and cluster of the samples. The heatmap plot of the FPKM values of all DEGs showed a clear separation between the pcDNA3.1-SP-D and control group, and a high consistency among three replicates of the same groups (**Figure 1C**). On comparison of DEGs among various samples, we found that 141 genes were significantly up-regulated and 92 genes were down-regulated (**Figure 1D**). All the results above supported a conclusion that SP-D shows a significantly higher capacity of up-regulating than down-regulating gene expression. We used GO analysis to better understand the role of SP-D and related genes. All molecular processes, including the inflammatory responses, immune responses, LPS responses (**Figures 1E, F****)**, extracellular space, and chemokine activity (**Supplementary Figure 1**) were related to SP-D in chondrocytes. The KEGG pathway analyses revealed that these genes were significantly related to PI3K-Akt signaling, TLR signaling, ECM-receptor interactions, and Rap1 signaling pathways (**Figures 1G, H**). These results indicated that SP-D regulates the expression of many genes associated with inflammatory and immune responses, TLR and PI3K-Akt signalings.

### *In Vivo* Transfection of Chondrocytes with rAAV Vectors Reduced SP-D Expression and Promoted Inflammatory Immune Responses in Joint Cartilage

**Figure 2:**
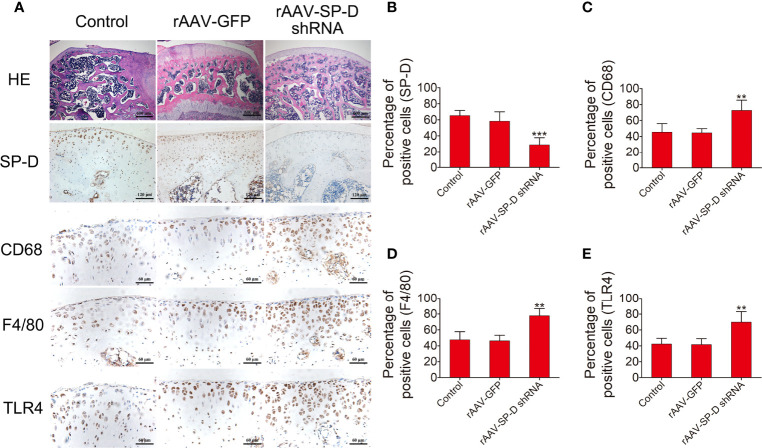
RAAV-mediated SP-D gene was transferred into the rat knee joints. **(A)** Rats were injected intra-articularly with rAAV encoding SP-D-specific shRNA for 10 consecutive days and sacrificed at the fourth week of the first injection. H&E staining and immunohistochemical staining of SP-D, CD68, F4/80, and TLR4 protein levels in cartilage were assessed following rAAV-GFP or rAAV-SP-D shRNA injection into the rat knee joints. The ratios of immunoreactive cells of SP-D **(B)**, CD68 **(C)**, F4/80 **(D)**, and TLR4 **(E)** were analyzed. Data were expressed as mean ± SEM (n = 5). ^**^P < 0.01 and ^***^P < 0.001 vs. rAAV-GFP group.

The intra-articular cartilage was treated with rAAV-SP-D shRNA to analyze its ability to change the SP-D expression in the cartilage. H&E staining showed that the morphology of articular cartilage did not change after SP-D silencing. Compared with the control animals, rAAV-SP-D shRNA-treated animals had reduced SP-D expression in the cartilage tissues (**Figures 2A, B**). Western blot analysis showed that a loss of SP-D in rAAV-SP-D shRNA-treated synovium and cartilage compared to control animals was observed (**Supplementary Figure 2**). Furthermore, we evaluated the changes in the expression of macrophage specific markers. The levels of CD63, F4/80 and TLR4 in the cartilage tissue treated with rAAV-SP-D shRNA were significantly increased as compared to the cartilage tissues in the control and rAAV-GFP-treated animals (**Figures 2C–E**). These results indicated that SP-D exerts a protective effect on articular cartilage by regulating the expression of immune-inflammatory proteins in articular cartilage tissue.

### Inhibition of the Infiltration of Synovial Immune Cells by SP-D and Its Chondroprotective Effects *In Vivo*

**Figure 3:**
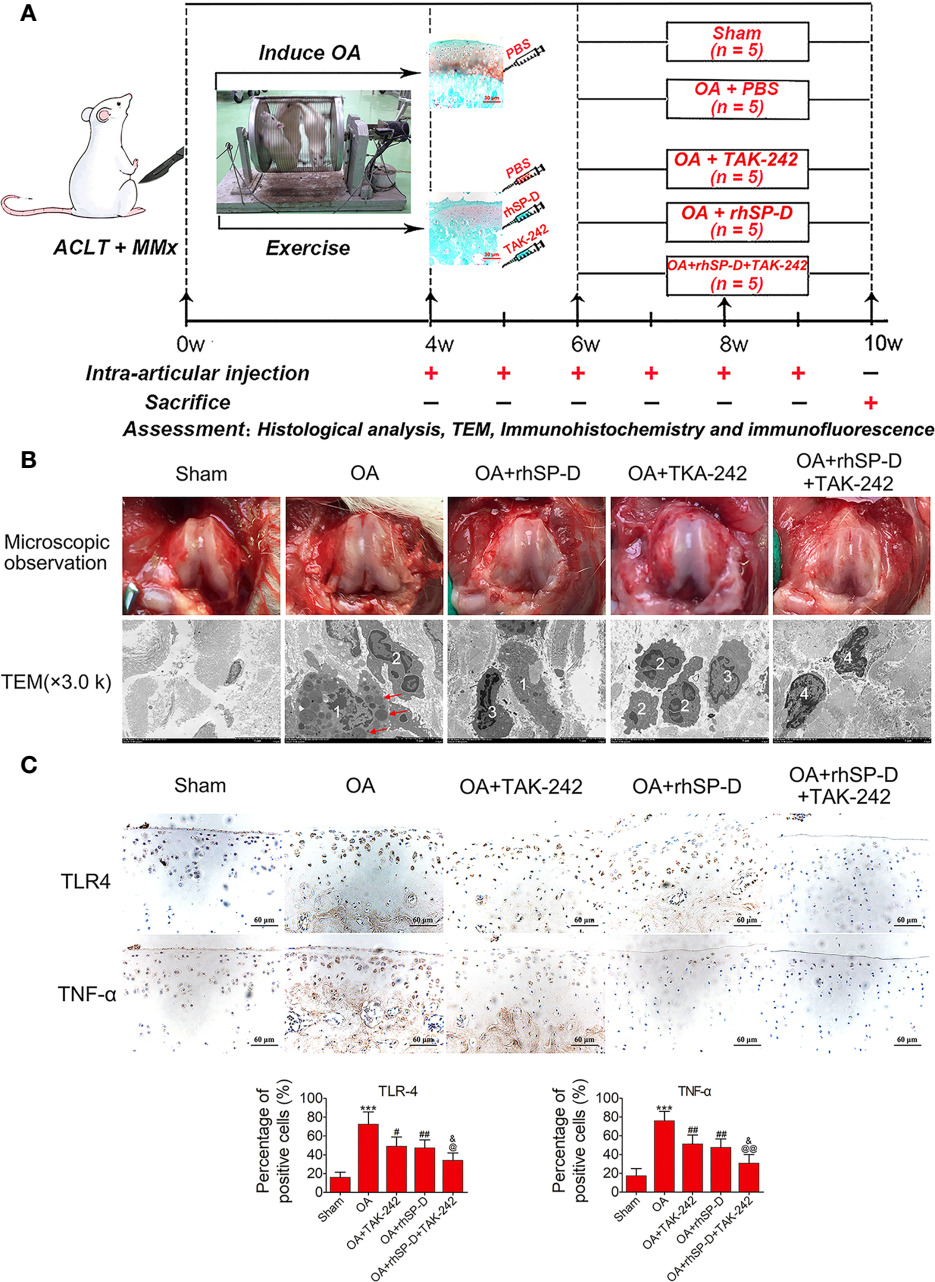
An overview of study timelines and suppression of inflammatory and immune responses by SP-D in the rat OA model. **(A)** The ACLT + MMx rats were put into an electronic rotator cage for 30 min per day as a means of inducing OA model beginning 1 week post-surgery. At 4 weeks post-surgery, animals were injected intra-articularly with different concentrations of rhSP-D and TAK-242 once per week. PBS was used as controls in sham and OA model animals. At 10 weeks post-operation, the animals were euthanized by cardiac exsanguination. Histological staining, immunohistochemistry, immunofluorescence, and TEM were used for detection. **(B)** Microscopic observation and synovial immune cells infiltration were assessed *via* TEM (3,000 ×). 1 = macrophage;2 = neutrophil;3 = lymphocyte;4 = synovial fibroblast;The red arrows represented lysosomes. **(C)** Immunohistochemical staining of TLR4 and TNF-α in ACLT + MMx-induced OA rats with the administration of rhSP-D and TAK-242. The ratios of immunoreactive cells were quantified. Data were expressed as mean ± SEM (n = 5). ^***^P < 0.001 vs. the sham-operated group; ^#^P < 0.05 and ^##^P < 0.01 vs. the OA-induction group; ^&^P < 0.05 vs. OA + rhSP-D group; ^@^P < 0.05 and ^@@^P < 0.01 vs. OA + TAK-242 group.

The timeline of OA modeling, intervention and sampling was shown in **Figure 3A**. Macroscopic observations were shown in **Figure 3B**. The femoral condyles cartilage in the sham group was smooth and free of osteophytes, while cartilage lesions developed on femoral condyles and the cartilage surface gloss was severely corroded in the OA-induction group. The cartilage tissue destruction was ameliorated after intra-articular injection of rhSP-D and TLR4 signaling antagonist (TAK-242). We evaluated the cellular immune infiltration in the synovial tissues using TEM (**Figure 3B**). The sham group animals showed collagen fibers with standard structures, while the OA-induction group animals showed irregular collagen fibers, and macrophages and neutrophils infiltration. This cellular immunity recruited many lysosomes and other immune-reactive substances. In the OA + rhSP-D group, the infiltration of macrophages was reduced, with a few neutrophils and lymphocytes. In the OA + TAK-242 + rhSP-D group, immune cells infiltration had significantly decreased, and the morphology of synovial fibroblasts could be observed.

To investigate the involvement of SP-D in the regulation of inflammatory responses, the inflammatory cytokines of TLR4 and TNF-α in the surgical OA model were determined by immunohistochemical analysis. The immunoreactivity expressed by TLR4 and TNF-α in the articular cartilage zone of the OA-induction group animals was significantly higher than that of the sham-operated animals (**Figure 3C**). Intra-articular injections of rhSP-D significantly inhibited the expression of TLR4 and TNF-α in cartilage derived from the rat OA model, while TAK-242 had a synergistic effect and enhanced the inhibitory effect of rhSP-D.

**Figure 4:**
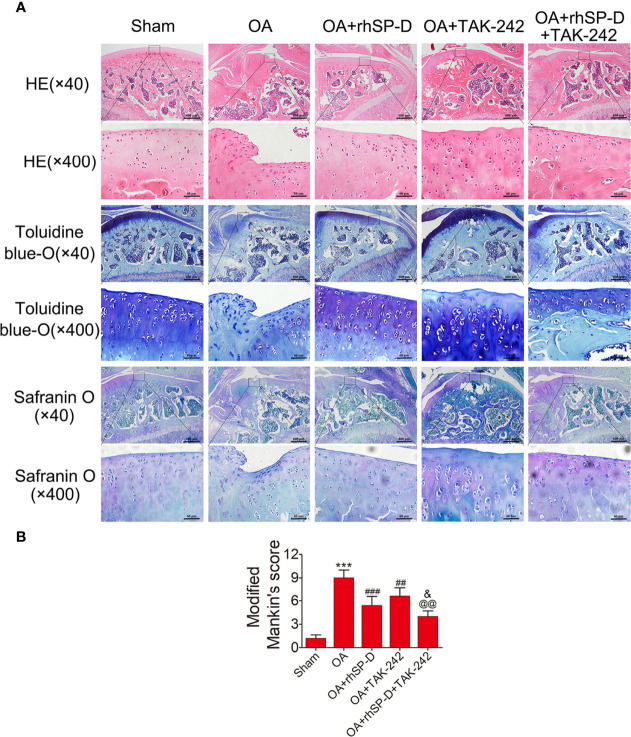
The rescue of cartilage degeneration by SP-D in rat OA model. **(A)** H&E, Safranin O and toluidine blue O-stained tissue histology in rat articular cartilage at 10 weeks post-surgery. **(B)** The modified Mankin scores were assigned to tissue samples. Data were expressed as mean ± SEM (n = 5). ^***^P < 0.001 vs. the sham-operated group; ^##^P < 0.01 and ^###^P < 0.001 vs. the OA-induction group; ^&^P < 0.05 vs. OA + rhSP-D group; ^@@^P < 0.01. vs. OA + TAK-242 group.

We studied the pathology of the surface layer of cartilage tissue in the slices stained with H&E, Toluidine blue-O and Safranin O stains. A certain degree of cartilage defects and loss of hyaline cartilage was observed in the OA-induction group (**Figure 4A**). The severity of cartilage degeneration in the rhSP-D and TAK-242 treatment groups was much lesser than that of the OA-induction group, as indicated by the cartilage thickness and surface regularity. rhSP-D and TAK-242 maintained cartilage tissues and avoided cartilage deterioration, as indicated by the low modified Mankin scores as compared with the OA-induction group (**Figure 4B**). The modified Mankin score of the OA + TAK-242 + rhSP-D group was significantly lower than that of the OA + rhSP-D group. These results indicated that SP-D was involved in the inhibition of synovial immune cells infiltration, and SP-D synergistically delayed cartilage degeneration along with TLR4 inhibitor TAK-242.

### SP-D Modulated Inflammatory Responses by Suppressing TLR4-Mediated PI3K/Akt Activation and NF-κB Signaling

**Figure 5:**
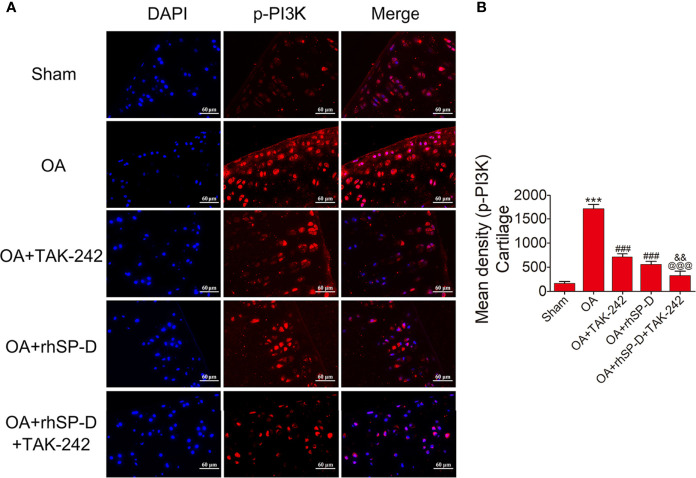
Suppression of TLR4-mediated PI3K signaling by SP-D treatment *in vivo*. **(A)** Immunofluorescence with an antibody to p-PI3K in articular cartilage from the ACLT + MMx-induced OA rats with rhSP-D and TAK-242 treatment at 10 weeks post-surgery. **(B)** The ratios of immunoreactive cells were quantified in articular cartilage according to immunofluorescence. Data were expressed as mean ± SEM (n = 5). ^***^P < 0.001 vs. the sham-operated group; ^###^P < 0.001 vs. the OA-induction group; ^&&^P < 0.01 vs. OA + rhSP-D group; ^@@@^P < 0.001 vs. OA + TAK-242 group.

To investigate whether SP-D could regulate PI3K/Akt and NF-κB p65 transcription, we examined the phosphorylation levels of PI3K, Akt and NF-κB p65 in the cartilage of ACLT + MMx surgically induced rat OA model by immunofluorescence (**Figures 5****–** **7**). The percentages of p-PI3K, p-Akt and p-p65-positive cells in the OA-induced group were significantly higher than those in the sham-operation group. The staining of p-PI3K, p-Akt and p-p65 were significantly concentrated in the cytoplasm and nucleus of chondrocytes in the cartilage of the OA-induced group. We found that rhSP-D reduced phosphorylation levels of PI3K, Akt and NF-κB p65 in cartilage derived from rats with surgically induced OA. We injected TLR4 inhibitor TAK-242, which inhibited the expression of p-PI3K, p-Akt and p-p65 in comparison with the OA-induced group. When we injected both rhSP-D and TAK-242 together, the inhibitory effect of rhSP-D was increased. Similar results were obtained when we evaluated the phosphorylation of PI3K, Akt and NF-κB p65 expression in synovial tissues (**Supplementary Figures 3**–**5**). Together, these data indicated that SP-D modulates inflammatory responses by suppressing TLR4-mediated downstream PI3K/Akt and NF-κB signalings.

**Figure 6:**
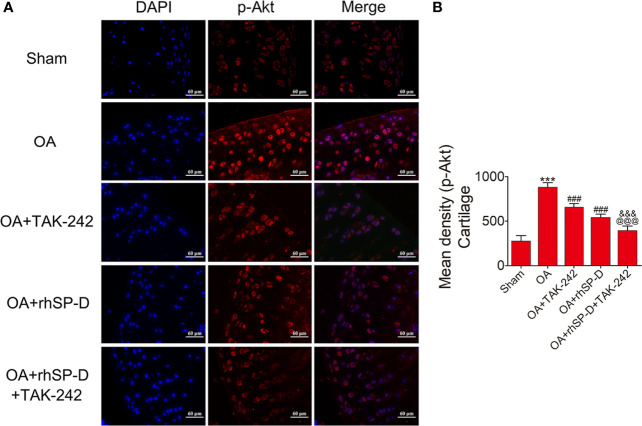
SP-D modulated TLR4-mediated Akt signaling *in vivo***. (A)** Immunofluorescence with an antibody to p-Akt in articular cartilage from the ACLT + MMx -induced OA rats with rhSP-D and TAK-242 treatment at 10 weeks post-surgery. **(B)** The ratios of immunoreactive cells were quantified in articular cartilage according to immunofluorescence. Data were expressed as mean ± SEM (n = 5). ^***^P < 0.001 vs. the sham-operated group; ^###^P < 0.001 vs. the OA-induction group; ^&&&^P < 0.001 vs. OA + rhSP-D group; ^@@@^P < 0.001 vs. OA + TAK-242 group.

**Figure 7:**
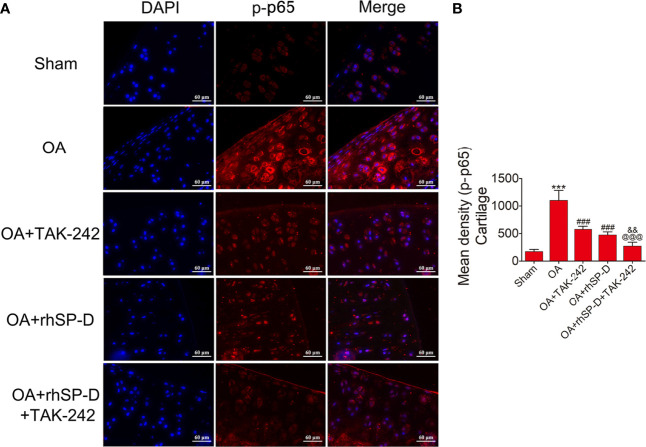
SP-D regulated TLR4-mediated NF-κB pathway *in vivo*. **(A)** Immunofluorescence with an antibody to p-p65 in articular cartilage from the ACLT + MMx-induced OA rats with rhSP-D and TAK-242 treatment at 10 weeks post-surgery. **(B)** The ratios of immunoreactive cells were quantified in articular cartilage according to immunofluorescence. Data were expressed as mean ± SEM (n = 5). ^***^P < 0.001 vs. the sham-operated group; ^###^P < 0.001 vs. the OA-induction group; ^&&^P < 0.01 vs. OA + rhSP-D group; ^@@@^P < 0.001 vs. OA + TAK-242 group.

### SP-D Reduced Chondrocyte Inflammatory Responses Induced by LPS

**Figure 8:**
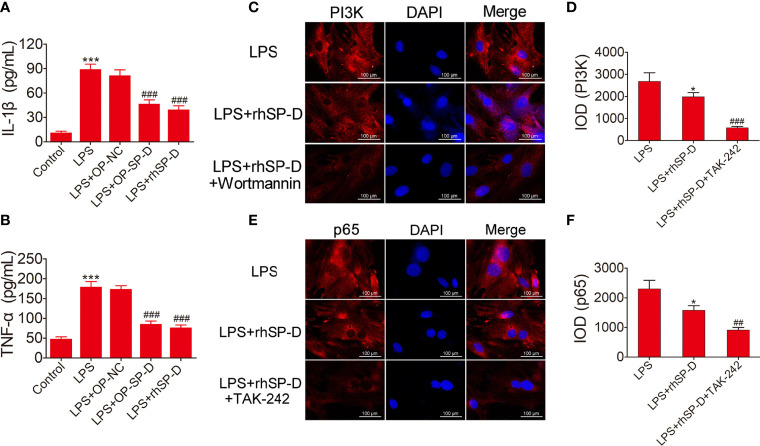
Suppression of inflammatory response and the PI3K and NF-κB pathways by SP-D treatment *in vitro*. **(A, B)** Chondrocytes were pre-incubated with rhSP-D and transfection of pcDNA3.1-SP-D plasmid for 2 h before LPS co-treatment for 24 h. The concentrations of IL-1β and TNF-α were determined by ELISA. Data were expressed as mean ± SEM (n = 3). ^***^P < 0.001 vs. control group; ^###^P < 0.001 vs. LPS group. **(C–F)** Immunofluorescence with antibodies to PI3K and p65 in chondrocytes, and the fluorescence images of PI3K and p65-Tracker Red in chondrocytes. The IOD was quantified according to immunofluorescence. Data were expressed as mean ± SEM (n = 3). ^*^P < 0.05 vs. LPS group; ^##^P < 0.01 and ^###^P < 0.001 vs. LPS + rhSP-D group.

To investigate the involvement of SP-D in the regulation of inflammatory responses, LPS-induced inflammatory cytokines in rat chondrocytes were determined by ELISA analysis. The levels of IL-1β and TNF-α in chondrocytes were monitored. As shown in **Figures 8A, B**, the expression levels of IL-1β and TNF-α were induced by LPS, while transfection of pcDNA3.1-SP-D plasmid in chondrocytes led to the reduction of IL-1β and TNF-α. Pretreated with rhSP-D significantly abolished the elevation of IL-1β and TNF-α induced by LPS. These data suggest that SP-D protected chondrocytes from LPS-induced inflammatory cell responses.

### SP-D Suppressed the TLR4-Mediated PI3K/Akt Activation and NF-κB Signaling in LPS-Stimulated Chondrocytes

**Figure 9:**
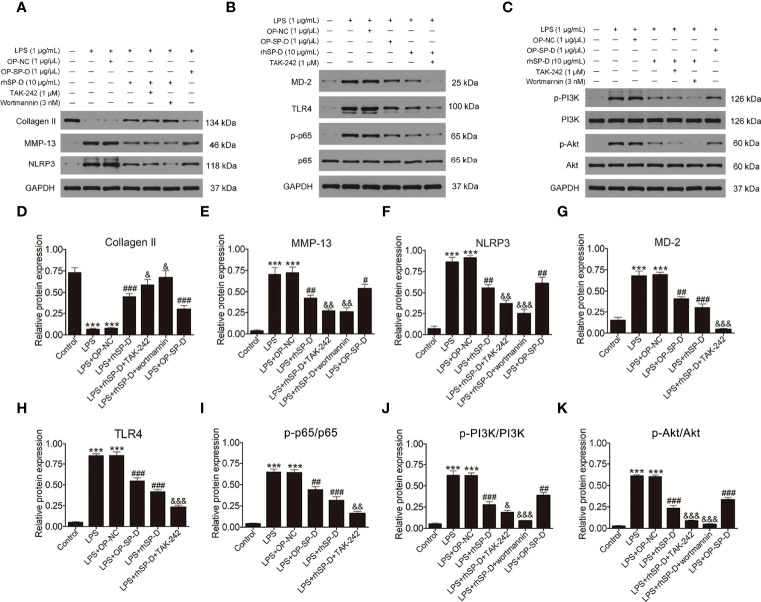
Effects of SP-D on inflammation and ECM by suppressing TLR4-mediated PI3k/Akt and NF-κB signalings in chondrocytes. **(A–C)** The protein expression of Collagen II, MMP-13, NLRP3, p-PI3K, PI3K, p-Akt, Akt, MD-2, TLR4, p-p65, and p65 were assessed *via* western blotting with GAPDH as a loading control. **(D–K)** The ratios of Collagen II, MMP-13, NLRP3, MD-2, and TLR4 to GAPDH, and p-PI3K/PI3K, p-Akt/Akt, p-p65/p65 were analyzed. Data were expressed as mean ± SEM (n = 3). ^***^P < 0.001 vs. the control group; ^##^P < 0.01 and ^###^P < 0.001 vs. the LPS group; ^&^P < 0.05, ^&&^P < 0.01 and ^&&&^P < 0.001 vs. the LPS + rhSP-D group. OP-SP-D, SP-D overexpressing plasmid; OP-NC, Negative control plasmid.

To determine whether SP-D inhibits TLR4 mediated PI3K/Akt activation and NF-κB p65 signaling, we treated chondrocytes with rhSP-D and/or TLR4 inhibitor TAK-242 and PI3K inhibitor wortmannin. Immunofluorescence staining was carried out to determine the expression of PI3K and NF-κB p65 in chondrocytes. As shown in **Figures 8C–F**, LPS promoted the expression of PI3K and p65, and rhSP-D inhibited the effects of LPS on the expression of PI3K and NF-κB p65 in chondrocytes. Moreover, pre-treatment with TAK-242 and wortmannin significantly enhanced the rhSP-D-mediated decrease in the densities of PI3K and p65 subunits. Western blot analysis showed that the expression levels of MMP-13 and NLRP3 in LPS-stimulated chondrocytes were significantly higher, while the level of collagen II was significantly lower than the control group (**Figures 9A, D–F**). We found that rhSP-D treatment and transfection of pcDNA3.1-SP-D plasmid significantly reduced the expression levels of MMP-13 and NLRP3, and increased the expression level of collagen II in LPS-induced chondrocytes, while TAK-242 and wortmannin enhanced the effect of rhSP-D on LPS-induced chondrocytes. The expression of TLR4, MD-2, p-p65, p-PI3K, and p-Akt increased in the LPS-stimulated group (**Figures 9B, C, G–K**). The administration of rhSP-D and transfection of pcDNA3.1-SP-D plasmid blocked the LPS-induced increase in TLR4, MD-2, p-p65, p-PI3K, and p-Akt. TAK-242 and wortmannin enhanced rhSP-D-mediated reduction in the expression of TLR4, MD-2, p-p65, p-PI3K, and p-Akt. Overall, SP-D inhibited the LPS-induced degradation of ECM and reduced LPS-induced chondrocyte inflammation by suppressing TLR4-mediated PI3K/Akt transcription factors and NF-κB activation.

### Molecular Binding Capacity of the SP-D and TLR4/MD-2 Complex

**Figure 10:**
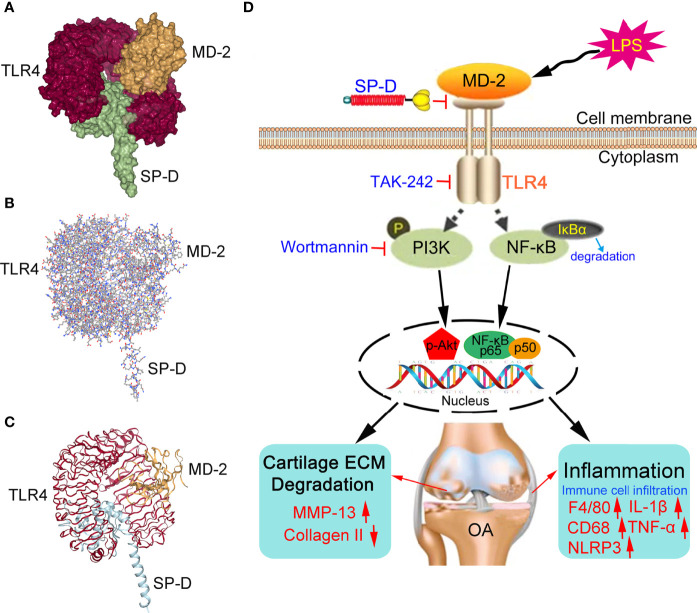
Proposed mechanisms of SP-D interference with OA chondrocyte inflammatory response and cartilage degradation. **(A–C)** The spatial filling model illustrated that SP-D was fully embedded in the inhibitory pocket of TLR4/MD-2 homodimer complex. The surface of SP-D binds to TLR4/MD-2 homodimer complex with average generalized born score of -25.03 kcal/mol and average poisson-boltzmann of -46.87 kcal/mol. **(D)** SP-D as a modulator of inflammatory response *via* inhibition of TLR4-mediated NF-κB activation and PI3K/Akt pathways.

Since SP-D inhibited the transcription factor of TLR4 and MD-2 receptors in chondrocytes, we performed docking calculations to explore the interactions between SP-D and TLR4/MD-2 complex (**Figures 10A–C**). As per the spatial filling model, the carbohydrate recognition domain of SP-D was completely inserted in the inhibitory pocket of the recombinant soluble forms of TLR4 and MD-2 homodimer complex. The surface layer of SP-D was fused with TLR4/MD-2 homodimer complex with average generalized born score of -25.03 kcal/mol and average poisson-boltzmann of -46.87 kcal/mol.

## Discussion

In this study, we demonstrated that SP-D suppresses OA-related immune responses and chondrocyte inflammation by inhibiting TLR4-mediated PI3K/Akt transcription and NF-κB activation. Firstly, transcriptome analysis revealed that SP-D overexpression broadly affects inflammatory response, immune response, and PI3K-Akt and TLR signaling in chondrocytes. Secondly, SP-D knockdown by rAAV injection resulted in the induction of inflammatory and innate responses in joint cartilage; however, these responses were not sufficient to promote disease progression. Finally, SP-D binds to TLR4/MD-2 complex to suppress TLR4-mediated PI3K/Akt and NF-κB signaling activation in osteoarthritic chondrocytes. All these data show that SP-D plays a functional role in OA and highlight its potential value in the treatment of OA.

The activation of the innate immune system is associated with tissue damage or chronic inflammation (22). The level of TLR4, an innate immune system mediator, rises in the cartilage tissues in surgically induced OA (23). Several strategies have been proposed to manipulate the signal transmission of the protein kinase in chondrocytes. Targeting TLRs and/or their downstream signaling pathways may inhibit the progression of OA. The modulation of SP-D involves various pathological inflammatory manifestations including subacute inflammation, which is the basis of this disease (24, 25). Previous reports indicate that the production of TLR4 and MD-2 complex is particularly important for LPS signaling, and the interaction between SP-D and the receptor complex may harm LPS signal transmission (26, 27).

To understand the molecular mechanisms underlying the therapeutic efficacy of SP-D against OA, we conducted a whole-genome transcriptome analysis using chondrocytes of rat cartilage. Gene expression analysis based on RNA-sequencing revealed that many genes are differentially controlled by SP-D. These genes were closely related to the critical pathways associated with inflammatory responses, cellular responses to LPS, immune responses, and cell proliferation. The analysis of the basic functions and pathways involving SP-D and those related to DEGs revealed that the OA pathways are highly involved in the PI3K-Akt signaling, ECM-receptor interactions, and TLR signaling. These findings direct for further research on the mechanism of SP-D underlying the modulation of inflammatory and immune responses of cartilage tissues.

To validate the anti-inflammatory effect of SP-D, we stimulated the chondrocytes with LPS and analyzed LPS-stimulated inflammatory responses. Among inflammatory cytokines, the interleukin family, particularly IL-1β and TNF-α, play a leading role in the pathological progression of OA (28, 29). During the pathological process of OA, inflammatory mediators, especially IL-1β, induce the release of other pro-inflammatory cytokines, thereby promoting the catabolic response and destroying the structure of the articular cartilage in chondrocytes. MMP-13 promotes the degradation of cartilage tissues, dissolves the components of ECM, and plays a role during the inflammation period of OA (30). The pro-inflammatory cytokines not only cause direct damage to cartilage, but also degrade the ECM and collagen II, destroying the joint cartilage. Therefore, molecules capable of targeting LPS-induced inflammation may have the potential to be therapeutic drug candidates for OA. In the present study, we found that SP-D suppressed the increase in MMP-13, NLRP3, IL-1β, and TNF-α in LPS-induced chondrocytes. The anti-inflammatory effect of SP-D was related to the inhibition of TLR4 expression. SP-D is known to bind to several carbohydrate ligands, including LPS from different strains of bacteria *via* its lectin domains. Further, LPS induced chondrocyte inflammation; SP-D pre-incubated with LPS inhibited LPS induced inflammation. The LPS-sensing receptor, TLR4, plays a pivotal role in cell survival and inflammation. Activation of TLR4 triggered the release of pro-inflammatory cytokines such as IL-1β and TNF-α, resulting in damage to the host cells. These findings partially suggest that SP-D plays a negative role in inflammatory responses and prevents the degradation of ECM by regulating the function of TLR4.

To explore the regulatory function of SP-D in innate immunity, we disrupted the expression of SP-D by injecting rAAV into the knee joints of rats. In the present study, we observed that SP-D disruption in all normal knee joints does not cause the deterioration of cartilage tissues in the long term. In addition, SP-D knockdown led to the increased expression of F4/80, CD68, TLR4 and other innate immune response proteins, indicating that endogenous SP-D is likely to modulate the immune responses in all normal cartilage tissues. Although we observed no structural changes and degeneration of articular cartilage during the onset and early development of OA, we found reduced expression of SP-D on the cartilage surface. The increased levels of inflammatory factors damaged the defense barrier of cartilage, leading to cartilage degeneration and the onset of OA. We reduced the level of SP-D in normal cartilage tissues through artificial intervention, including an intra-articular injection of rAAV. The expression of inflammatory factors in cartilage tissue increased, and the natural immune function of cartilage was gradually affected. Under such conditions, we assumed that even minor triggers can lead to the onset of OA. Meanwhile, SP-D suppressed the hyperplasia of the synovial membrane and infiltration of immune cells. These results suggested that endogenous SP-D promotes innate immunity in rat articular cartilage and synovial tissues. Absence of SP-D in the knocked-down animals is associated with higher intrinsic pro-inflammatory status. Therefore, the higher level of CD68, F4/80 and TLR4 could be a consequence of many factors other than SP-D knockdown. These findings are consistent with previous reports, demonstrating that endogenous SP-D manipulates the cellular immunomodulation checkpoint in chondrocytes, joint articular cartilage, and synovial membrane (31).

The inflammatory microenvironment of the OA joint is found to be orchestrated by macrophages, neutrophils, and multiple inflammatory cytokines. Previous studies have demonstrated the importance of macrophages and neutrophils in driving inflammatory and destructive responses in OA (32, 33). These results indicated there is a triggering low-grade inflammatory cycle that leads to the infiltration of macrophages and neutrophils and further induce structural changes in the synovial tissues of OA rats. We established the rat OA model and injected TLR4 inhibitor TAK-242 to investigate whether SP-D participates in the inhibition of cartilage and synovial inflammation by inactivating TLR4 signaling. Surgical OA modeling leads to the decrease of SP-D activity, promotes the infiltration of immune cells in the synovial tissues and activates TLR4 signaling, leading to the release of IL-1β and TNF-α. SP-D reduced inflammatory cytokines and immune cells (neutrophils and macrophages) *via* modulating the TLR4 signaling. Based on these findings, we postulate that SP-D is a barrier to cartilage surfactant homeostasis and inhibits the innate immune responses and maintains the stability of the joint structure.

The endogenous molecules released from the injured tissues and inflammatory cells activate pro-inflammatory responses by interacting with TLR4. The analysis of the KEGG pathway revealed that these genes were significantly related to PI3K-Akt signaling and TLR signaling. We selected the PI3K-Akt signaling pathway that ranked high and was closely related to the regulation of inflammation. The PI3K/Akt signaling pathway is one of the most crucial cell survival pathways that participates in the modulation of inflammatory responses, cellular activation, and apoptosis (34, 35). The induction of PI3K/Akt signaling reduces the pro-inflammatory response of the NF-κB pathway (36). LPS stimulates TLR4 and promotes the expression of cell survival genes by activating the NF-κB pathway (37, 38). Pre-treatment with a moderate amount of LPS protects human dendritic cells or myocytes from apoptosis *via* PI3K/Akt and NF-κB dependent sensory system of TLR4 (39, 40). In the present study, pretreatment with LPS enhanced the activation of PI3K/Akt and NF-κB signaling in chondrocytes. These manifestations were abrogated by SP-D, and TAK-242 enhanced the inhibitory effect of SP-D. These findings suggest that crosstalk may exist between TLR4/NF-κB and PI3K/Akt signaling pathways in the regulation of inflammation in chondrocytes pretreated with LPS. However, despite proving that SP-D could exert its anti-inflammatory effects *via* the PI3K/Akt and NF-κB signalings, its potential upstream and downstream cascading effects should be studied further. Previous studies revealed that TLR4 signaling is currently one of the most widely studied upstream cascades, and the concentration of TLR4 in PI3K/Akt and NF-κB activation and that the OA development has received increasing attention (41, 42). We further explored whether SP-D targets the TLR4/MD-2 complex. TLR4/MD-2/LPS complex formation results in the intracellular domain of TLR4 being cascaded. SP-D exerted anti-inflammatory activities by affecting the proper assembly of the TLR4/MD-2/LPS ternary complex, the activation of which is associated with OA. We simulated the potential interaction between SP-D and TLR4/MD-2 complex using docking approaches, and found that SP-D specifically binds to the MD-2 inhibitory pocket and interferes with the formation of the TLR4/MD-2 complex. Using docking approaches, we further simulated the underlying mechanism of SP-D interacting with TLR4/MD-2 and found that SP-D specifically interferes with the formation of the TLR4/MD-2 complex. Therefore, SP-D binds to the TLR4 and MD-2 complex and suppresses inflammation and innate immune responses by decreasing the PI3K/Akt mediated activation of the TLR4 receptor and NF-κB transcription factors (**Figure 10D**).

In conclusion, SP-D suppresses LPS-induced inflammatory and innate responses in rat articular chondrocytes by inhibiting TLR4-mediated PI3K/Akt activation and NF-κB signalings. Therefore, SP-D may be considered as a potential therapeutic intervention to prevent the progression of OA. Meanwhile, SP-D knockout mice should be used in future study, which could further demonstrate the critical role of SP-D in OA.

## Data Availability Statement

The datasets presented in this study can be found in online repositories. The name of the repository and accession number can be found below: NCBI Sequence Read Archive; PRJNA836943.

## Ethics Statement 

This study was approved by the Animal Care and Use Committee of Medical School, Wuhan University (WDRM 20160104). Written informed consent was obtained from the owners for the participation of their animals in this study.

## Author Contributions

YZ, HJ, YuZ, and GH initiated the study, designed experiments and wrote the paper. YuZ, MD, YL, and YM performed experiments. YZ, JM, and XS analyzed and interpreted the data. YZ, YL, and SL reviewed the manuscript and wrote the paper. All authors have critically reviewed the manuscript and approved the final manuscript.

## Funding

This project was funded by National Natural Science Foundation of China (grant number: 81802203), Fundamental Research Funds for the Central Universities (grant number: 2042018kf0123), Guiding Fund of Renmin Hospital of Wuhan University (grant number: RMYD2018M43).

## Conflict of Interest

The authors declare that the research was conducted in the absence of any commercial or financial relationships that could be construed as a potential conflict of interest.

## Publisher’s Note

All claims expressed in this article are solely those of the authors and do not necessarily represent those of their affiliated organizations, or those of the publisher, the editors and the reviewers. Any product that may be evaluated in this article, or claim that may be made by its manufacturer, is not guaranteed or endorsed by the publisher.

## Supplementary Material

Supplementary Table 1 Summary of sample names, description, the RNA-seq sequencing information, and mapping results in each sample.

Supplementary Figure 1 DEGs were annotated to GO database to detect the gene function. **(A–D)** GO analysis showed that the biological processes such as regulation of ‘extracellular space’, ‘protein binding’ and ‘chemokine activity’ were associated with SP-D in chondrocytes.

Supplementary Figure 2 RAAV-mediated SP-D gene was transferred into the rat knee joints. **(A)** SD rats received an intra-articular injection of rAAV encoding SP-D-specific shRNA for SP-D or GFP into the knee for 10 consecutive days. SP-D expression in synovium and cartilage was assessed *via* western blotting. **(B)** The ratios of SP-D in synovium and cartilage were analyzed. Data were expressed as mean ± SEM (n = 3). ***P < 0.001 versus the rAAV-GFP group.

Supplementary Figure 3 Suppression of TLR4 mediated PI3K signaling by SP-D treatment in OA synovium. **(A)** Immunofluorescence with an antibody to p-PI3K in synovium. **(B)** The ratios of immunoreactive cells were quantified in synovium according to immunofluorescence. Data were expressed as mean ± SEM (n = 5). ^###^P < 0.001 vs. the OA-induction group; ^&&&^P < 0.001 vs. OA + rhSP-D group.

Supplementary Figure 4 Suppression of TLR4 mediated Akt signaling by SP-D treatment in OA synovium. **(A)** Immunofluorescence with an antibody to p-Akt in synovium. **(B)** The ratios of immunoreactive cells were quantified in synovium. Data were expressed as mean ± SEM (n = 5). ^###^P < 0.001 vs. the OA-induction group; ^&&&^P < 0.001 vs. OA + rhSP-D group.

Supplementary Figure 5 Suppression of TLR4 mediated NF-κB signaling by SP-D treatment in OA synovium. **(A)** Immunofluorescence with an antibody to p-p65 in synovium. **(B)** The ratios of immunoreactive cells were quantified in synovium. Data were expressed as mean ± SEM (n = 5). ^##^P < 0.01 vs. the OA-induction group; ^&&^P < 0.01 vs. OA + rhSP-D group.
