# Supplementary figures and images for: Innate/Inflammatory Bioregulation of Surfactant Protein D Alleviates Rat Osteoarthritis by Inhibiting Toll-Like Receptor 4 Signaling
Source: Front Immunol. 2022 Jul 5;13:913901. doi: 10.3389/fimmu.2022.913901 (PMC9294227; doi:10.3389/fimmu.2022.913901)

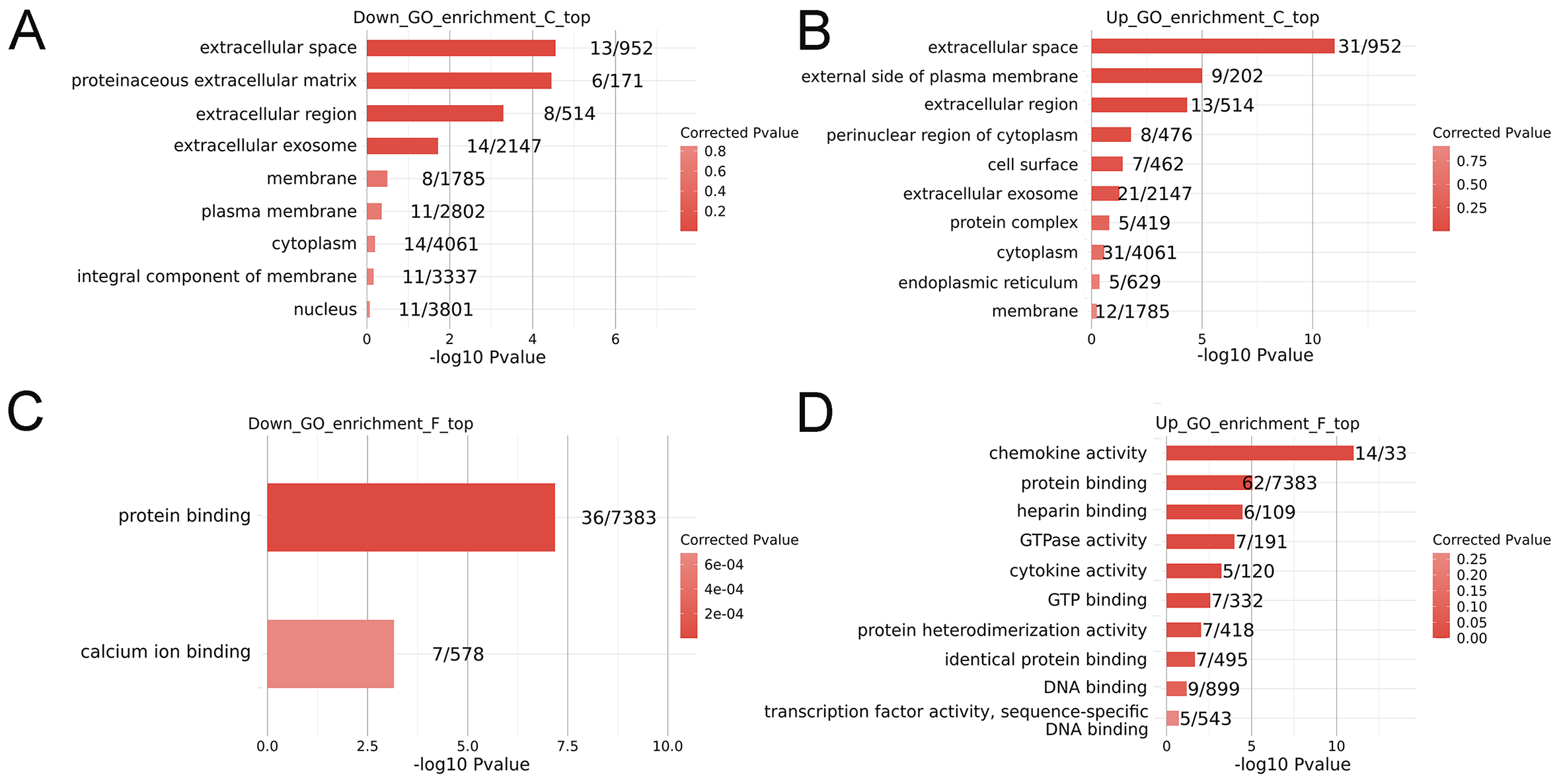

Supplement: Supplementary Figure 1 — DEGs were annotated to GO database to detect the gene function. (A–D) GO analysis showed that the biological processes such as regulation of ‘extracellular space’, ‘protein binding’ and ‘chemokine activity’ were associated with SP-D in chondrocytes. [file Image_1.tif]

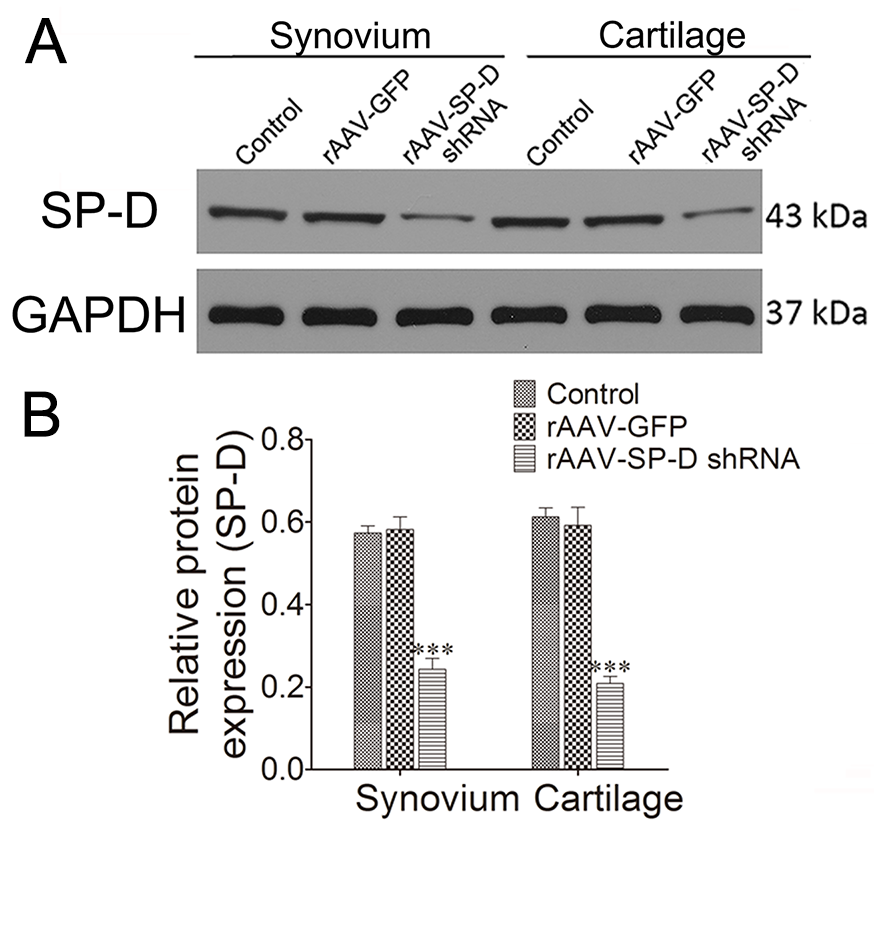

Supplement: Supplementary Figure 2 — RAAV-mediated SP-D gene was transferred into the rat knee joints. (A) SD rats received an intra-articular injection of rAAV encoding SP-D-specific shRNA for SP-D or GFP into the knee for 10 consecutive days. SP-D expression in synovium and cartilage was assessed via western blotting. (B) The ratios of SP-D in synovium and cartilage were analyzed. Data were expressed as mean ± SEM (n = 3). ***P < 0.001 versus the rAAV-GFP group. [file Image_2.tif]

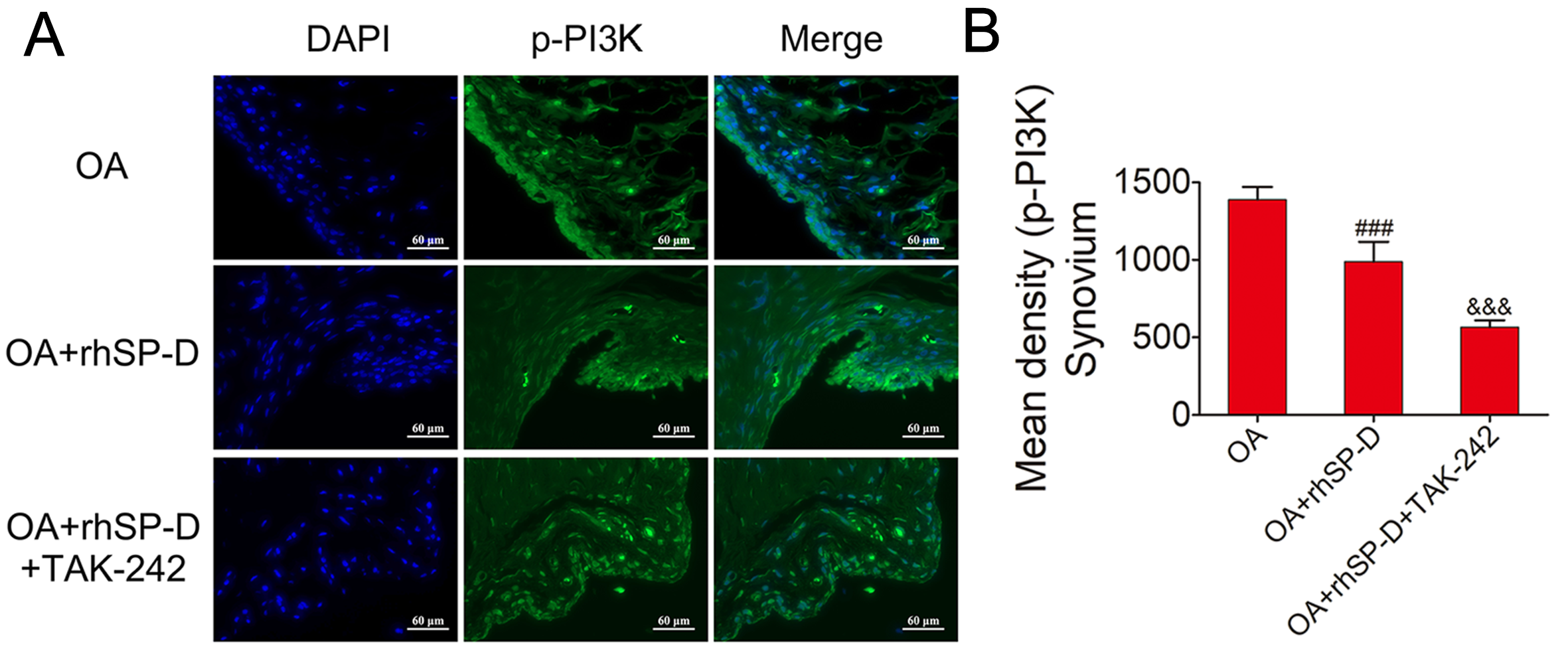

Supplement: Supplementary Figure 3 — Suppression of TLR4 mediated PI3K signaling by SP-D treatment in OA synovium. (A) Immunofluorescence with an antibody to p-PI3K in synovium. (B) The ratios of immunoreactive cells were quantified in synovium according to immunofluorescence. Data were expressed as mean ± SEM (n = 5). ###P < 0.001 vs. the OA-induction group; &&&P < 0.001 vs. OA + rhSP-D group. [file Image_3.tif]

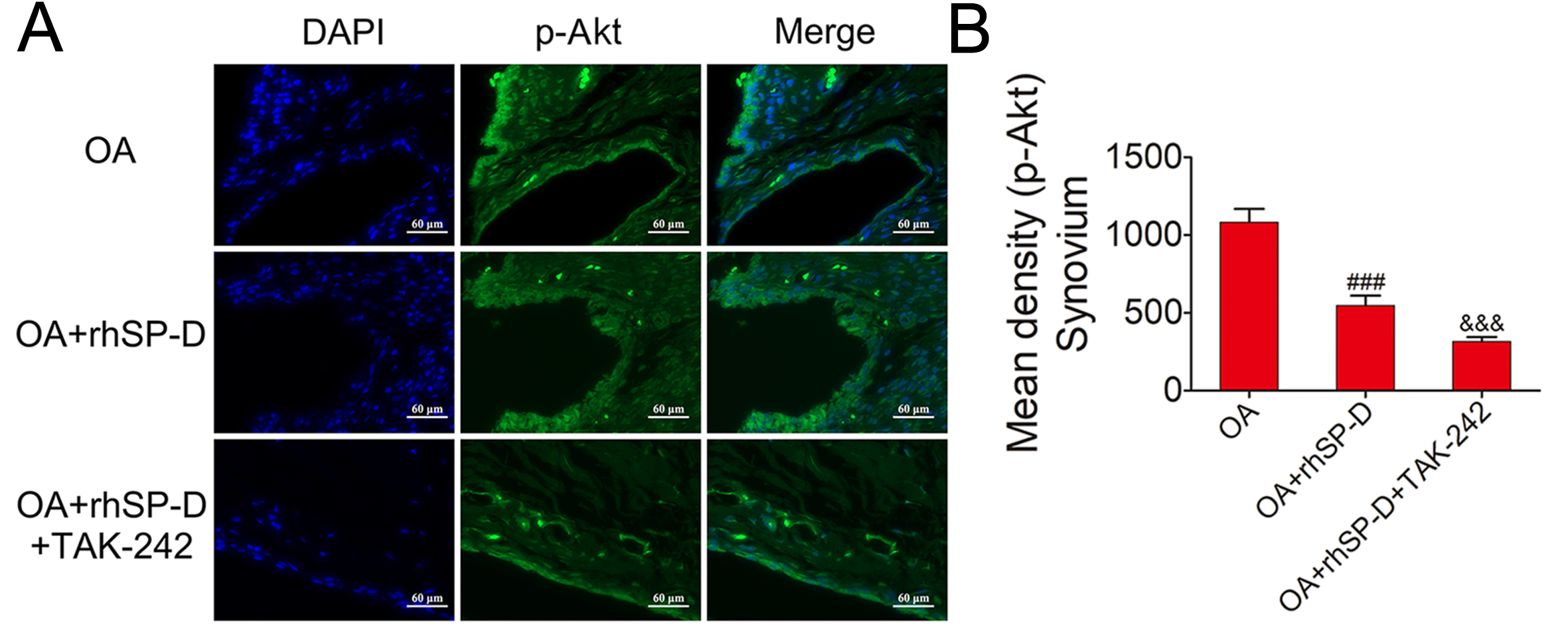

Supplement: Supplementary Figure 4 — Suppression of TLR4 mediated Akt signaling by SP-D treatment in OA synovium. (A) Immunofluorescence with an antibody to p-Akt in synovium. (B) The ratios of immunoreactive cells were quantified in synovium. Data were expressed as mean ± SEM (n = 5). ###P < 0.001 vs. the OA-induction group; &&&P < 0.001 vs. OA + rhSP-D group. [file Image_4.tif]

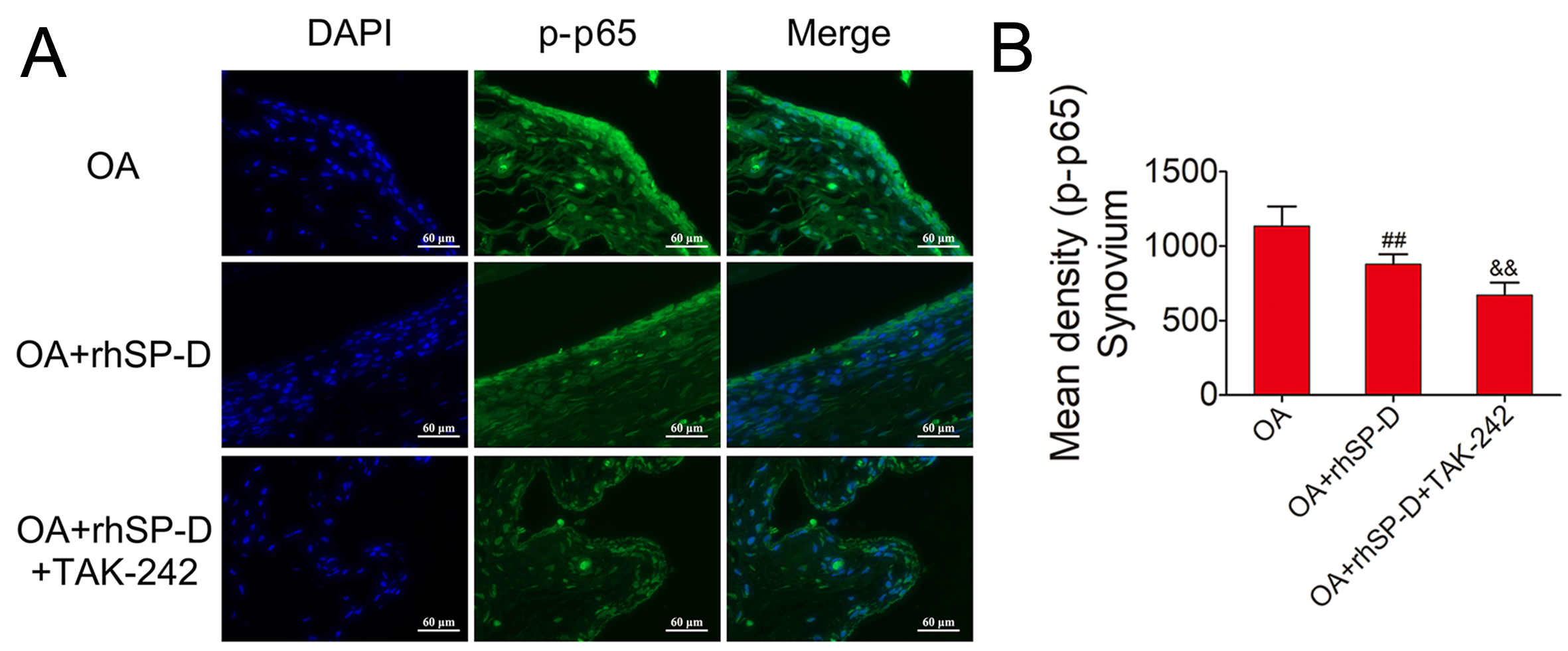

Supplement: Supplementary Figure 5 — Suppression of TLR4 mediated NF-κB signaling by SP-D treatment in OA synovium. (A) Immunofluorescence with an antibody to p-p65 in synovium. (B) The ratios of immunoreactive cells were quantified in synovium. Data were expressed as mean ± SEM (n = 5). ##P < 0.01 vs. the OA-induction group; &&P < 0.01 vs. OA + rhSP-D group. [file Image_5.tif]
